# Real-time foot clearance biofeedback to assist gait rehabilitation following stroke: a randomized controlled trial protocol

**DOI:** 10.1186/s13063-019-3404-6

**Published:** 2019-05-31

**Authors:** Rezaul Begg, Mary P. Galea, Lisa James, W. A. Tony Sparrow, Pazit Levinger, Fary Khan, Catherine M. Said

**Affiliations:** 1 Institute for Health and Sport (IHES), Victoria University, Footscray Park Campus (Room PB307), PO Box 14428, Melbourne, VIC 8001 Australia; 2 Royal Melbourne Hospital, Australian Rehabilitation Research Centre, Parkville, Victoria 3010 Australia; 3 The University of Melbourne, Department of Physiotherapy, 34-54 Poplar Road, Parkville, Victoria 3052 Australia; 4 National Ageing Research Institute Ltd., Clinical Gerontology Division, Royal Melbourne Hospital, PO Box 2127, Melbourne, VIC 3050 Australia; 5 Austin Health, Heidelberg Repatriation Hospital, PO Box 5444, Heidelberg West, Victoria 3084 Australia; 6 Western Centre for Health Research and Education, Sunshine Hospital, Western Health, Furlong Rd, St Albans, VIC 3021 Australia; 7 Australian Institute of Musculoskeletal Science, St Albans, Australia; 8 The University of Melbourne, Department of Medicine, Melbourne, Australia

**Keywords:** Gait, Stroke, Biofeedback, Falls, Tripping, Minimum toe clearance (MTC)

## Abstract

**Background:**

The risk of falling is significantly higher in people with chronic stroke and it is, therefore, important to design interventions to improve mobility and decrease falls risk. Minimum toe clearance (MTC) is the key gait cycle event for predicting tripping-falls because it occurs mid-swing during the walking cycle where forward velocity of the foot is maximum.

High forward velocity coupled with low MTC increases the probability of unanticipated foot-ground contacts. Training procedures to increase toe-ground clearance (MTC) have potential, therefore, as a falls-prevention intervention. The aim of this project is to determine whether augmented sensory information via real-time visual biofeedback during gait training can increase MTC.

**Methods:**

Participants will be aged > 18 years, have sustained a single stroke (ischemic or hemorrhagic) at least six months previously, able to walk 50 m independently, and capable of informed consent. Using a secure web-based application (REDCap), 150 participants will be randomly assigned to either no-feedback (Control) or feedback (Experimental) groups; all will receive 10 sessions of treadmill training for up to 10 min at a self-selected speed over 5–6 weeks. The intervention group will receive real-time, visual biofeedback of MTC during training and will be asked to modify their gait pattern to match a required “target” criterion. Biofeedback is continuous for the first six sessions then progressively reduced (faded) across the remaining four sessions. Control participants will walk on the treadmill without biofeedback. Gait assessments are conducted at baseline, immediately following the final training session and then during follow-up, at one, three, and six months. The primary outcome measure is MTC. Monthly falls calendars will also be collected for 12 months from enrolment.

**Discussion:**

The project will contribute to understanding how stroke-related changes to sensory and motor processes influence gait biomechanics and associated tripping risk. The research findings will guide our work in gait rehabilitation following stroke and may reduce falls rates. Treadmill training procedures incorporating continuous real-time feedback may need to be modified to accommodate stroke patients who have greater difficulties with treadmill walking.

**Trial registration:**

Australia New Zealand Clinical Trials Registry, ACTRN12617000250336. Registered on 17 February 2017.

## Background

Stroke affects > 60,000 Australians every year, with 50% unable to walk one week following the event [1]. Impaired walking impacts independence by reducing the ability to perform everyday activities and limiting community participation [2, 3]. Falls risk is significantly higher in people with chronic stroke [4] and approximately 50% of people living at home after a stroke will fall within 12 months [9], with up to half sustaining multiple falls. Furthermore, in community-dwelling people with stroke, up to 77% of falls occurred during walking. While there has been considerable research investigating falls risk management for older people generally, high-risk groups, such as those who have had a stroke, have not been extensively studied with respect to targeted falls prevention. Traditional exercise-based falls-prevention programs are useful for the general older adult community but are not effective in people with stroke. For example, Batchelor et al. found that a multifactorial intervention including a home-based balance and strength program did not reduce falls in people with stroke [10]. Another study confirmed that a group- and home-based exercise program incorporating balance and strength training did not reduce falls [11]. This suggests that alternative, targeted treatments to reduce falls risk in people with stroke are urgently needed.

Stroke adversely affects sensorimotor function and muscle strength, inhibiting the capacity to activate appropriate muscles and increasing the risk of contact between the foot and either the supporting surface or objects on it. Said et al. [15] found, for example, that stroke participants who had difficulty in stepping over small obstacles (4 cm high) had greater falls rates. The key gait variable for predicting tripping-falls is minimum toe clearance (MTC), an event mid-swing in the walking cycle [5–8]. Low MTC increases the probability of unanticipated foot-ground contacts [7]. Given that tripping directly results from unsuccessful toe clearance, previous research with both young and older populations has focused on toe trajectory control during walking [6–8, 12–14]. Training individuals to increase MTC, therefore, has potential as a falls-prevention intervention.

The aim of this project is to determine whether real-time biofeedback of toe clearance during gait training can significantly minimize tripping risk in people with stroke. We will test the efficacy of real-time biofeedback as an intervention to increase MTC using a randomized controlled trial (RCT) design incorporating both a training or “acquisition” phase with biofeedback. Retention tests will be conducted to confirm *learning*, as demonstrated by the longer-term or “relative permanence” of the targeted behavior.

The primary objective is to determine whether real-time biofeedback of MTC during gait training will significantly increase MTC in people with stroke. We will also determine whether changes in MTC achieved on a treadmill transfer to overground walking. It is hypothesized that, compared to no-biofeedback training, visual biofeedback of foot clearance parameters during gait training will significantly increase toe-ground clearance (MTC) and MTC during biofeedback training will be retained in the longer term. It is also hypothesized that increases in MTC demonstrated in treadmill training will transfer to overground walking, such that tripping-risk in people with stroke is significantly reduced.

## Methods/Design

**Fig. 1:**
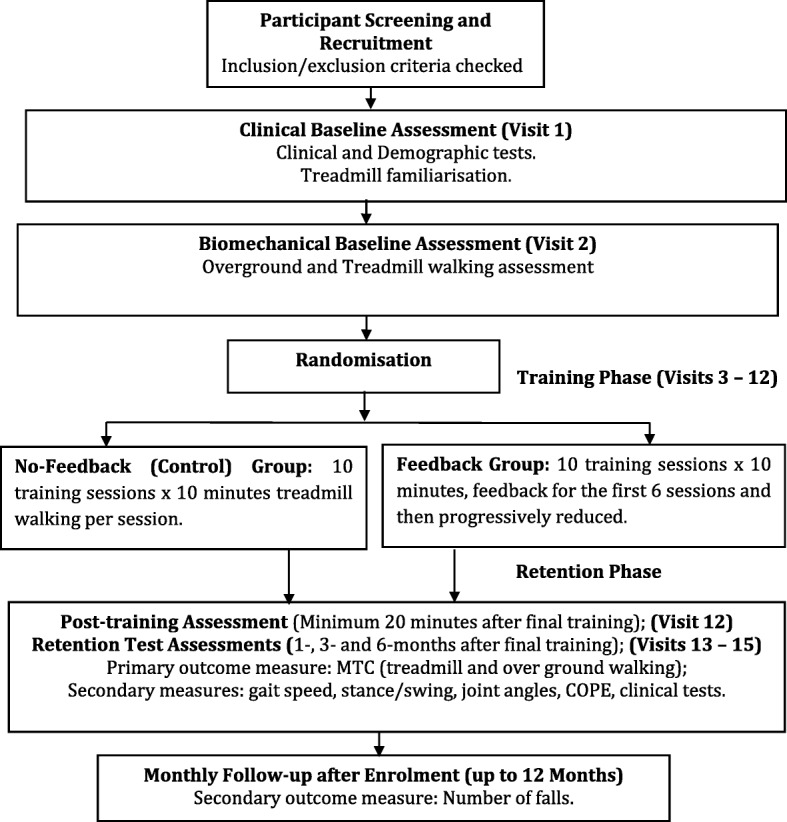
Study design flowchart

This single-blinded parallel group RCT with 1:1 randomization will assess the effects of biofeedback on MTC following gait training. It will conform to CONSORT guidelines and has been registered on the Australian and New Zealand Clinical Trials Registry (ANZCTR): ACTRN12617000250336. Ethics approval for the study was obtained from Austin Health, Melbourne Health, and Victoria University Human Research Ethics Committees. Informed consent procedures include the provision that participants clearly understand that they are free to withdraw at any time without providing a reason. The study design flowchart is shown in Fig. 1.

### Patient population

#### Eligibility criteria for participants

Participants will be aged > 18 years, have sustained a single stroke (ischemic or hemorrhagic) at least six months previously, able to walk 50 m independently with or without a single point stick, and capable of providing informed consent. Exclusion criteria are: (1) an ankle foot orthosis; (2) neurological, orthopedic, cardiac, respiratory, or other medical conditions in addition to stroke that impact their ability to walk on a treadmill; (3) body mass > 158 kg (due to the weight limit of the harness); and (4) visual problems or severe visual-spatial neglect. Participants will not be receiving physiotherapy for their walking or lower limbs while enrolled in the study.

#### Participant recruitment study settings

Recruitment sites include Heidelberg Repatriation Hospital (Austin Health), the Royal Melbourne Hospital (Royal Park Campus), and Western Health. Interested parties can also self-refer in response to advertising. Gait assessments and training are conducted at the Heidelberg Repatriation Hospital (Department of Physiotherapy) and the Victoria University (Footscray Park) Biomechanics Laboratory.

### Randomization

Randomization to participant group will be performed by the project coordinator using a secure, web-based Research Electronic Data Capture (REDCap) tool hosted at Victoria University [26]. The participant group allocation will then be e-mailed to the intervention therapist with the subject heading Randomization of (Participant ID). Participants will be coded and only available to authorized project personnel.

### Blinding

Clinical physiotherapists will be involved in assessment but blinded to group allocation and training sessions. Blinded clinical physiotherapists will conduct clinical assessments and collect falls-related data. Biomechanical gait assessors who conduct the movement analysis are, similarly, blinded to group allocation to ensure that all assessment personnel are blinded as to the participant’s group assignment. Physiotherapists and biomechanists delivering the gait training are required to know the patient’s group assignment.

### Intervention

All participants, both controls and those in the feedback intervention group, will walk on the treadmill following an identical schedule of training sessions. Self-selected walking speed will be determined following treadmill familiarization with a physiotherapist during the first clinical assessment visit. This self-selected walking speed will be recorded and each treadmill walking session will be conducted at the same walking speed. All participants wear a safety harness when treadmill walking and continue for up to 10 min with rest breaks as required. Previous research by the authors had determined 10 min to be comfortable maximum for this population. Participants will wear the same comfortable shoes for all walking activities.

Participants randomized to the intervention group undertake biofeedback gait-training using a real-time display of the affected limb’s swing phase trajectory, with toe clearance and the associated MTC event clearly shown. Motion analysis markers will be attached to the shoe and other body segments to calculate toe clearance parameters in real-time as per the established protocol [6]. *Target* MTC will be calculated as baseline MTC plus 1 SD. The feedback group will be asked to move their affected limb such that MTC falls between target MTC mean + 0.5SD and target MTC-0.5SD, projected as parallel lines on a screen in front of the treadmill [16, 17]. If baseline MTC cannot be calculated, target boundaries will be set using the *maximum* toe clearance (TC) with instructions to control foot motion within that target band. Borg Ratings of Perceived Exertion will be recorded after each session.

Biofeedback is presented for the first six sessions then progressively reduced (faded) across the remaining four sessions. During fading, one-third of the initial feedback will be available at the beginning of session 7, one-third in the middle for session 8, one-third at the end of session 9, and, finally, one-10th at the beginning and one-10th at the end of session 10. As indicated above, the control group will walk under the same conditions without either feedback or any instructions concerning their gait control. Adherence to the gait training program will be reflected in the number of training sessions successfully completed and the research team will monitor any non-adherence.

### Outcome measures

#### Assessments

A clinical assessment is conducted on the first visit (baseline) in order to describe the study population. Clinical assessments for global function, lower limb strength, gait speed, and falls risk are repeated following the final training session and at one, three, and six months after training. Clinical measures undertaken by the research physiotherapists will include:

- Functional Independence Measure (FIM) [18], a measure of global function;
- Timed Chair Stand Test [20], an indicator of lower limb strength;
- Timed 10-m Walk Test [19], an assessment of gait speed;
- Step Test [21], a balance measure that identifies stroke patients with a risk of falls;
- Sensory Testing (light touch, pinprick, and sensory extinction);
- Star cancellation test; to screen for visual-spatial neglect [27];
- Stroke Rehabilitation Assessment of Movement (STREAM) [22], measures voluntary movement and mobility in people with stroke;
- Tardieu scale [23], to assess spasticity in gastrocnemius and soleus;
- Six-minute walk test [24], a measure of walking endurance;
- Falls Risk for Older People in the Community (FROP-Com). The FROP-Com has previously been used to identify falls risk in people with stroke [10].

Gait assessment trials are conducted at baseline, after the final training session (a minimum of 20 min following final training) and one, three, and six months later. These gait data are collected during treadmill and overground walking by biomechanics personnel blinded to group allocation. During overground walking assessments (before and after training) participants walk at preferred speed along an 8-m walkway. Kinematic (position/time) data are captured using a three-dimensional motion analysis system (Optotrak®, NDI, Canada) with clusters of infrared markers fixed to the toe of the shoe and the shank, thigh, and pelvis segments. Bony landmarks on the foot, ankle, knee, and hip are identified using virtual markers and used with the clusters in order to construct a model of the joints and segments. This enables kinematic data, including joint angles, to be calculated. Foot plantar pressures and center of pressure excursion (COPE) are captured to assess gait stability using the Pedar foot pressure insole (Novel, Germany), an in-shoe system that measures plantar pressure distribution within the shoe.

#### Primary outcomes

The primary outcome variables from the motion analysis, MTC magnitude, and variability, will be assessed using biomechanical assessment data at baseline and after training, which occurs immediately following the final training session after a minimum of 20 min rest. Retention MTC data at one, three, and six months for the affected limb will determine whether the target MTC, i.e. baseline MTC plus 1 SD, has been maintained after visual biofeedback training has concluded. The precise probability of tripping due to foot-ground contact will be computed by statistically modelling the MTC histogram [7].

#### Secondary outcomes

**Fig. 2:**
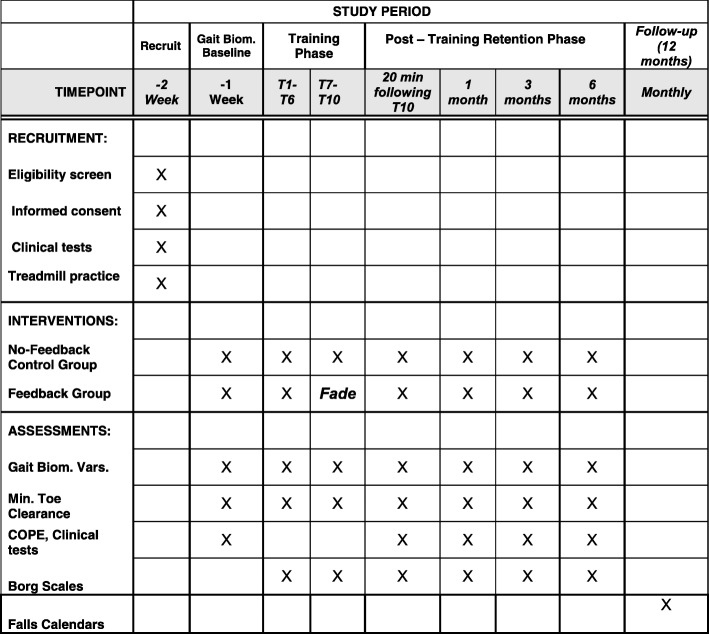
SPIRIT study timeline. T1 to T10 gait training sessions of (up to) 10 min treadmill walking. Feedback (FB) group given visual feedback display of minimum toe clearance (MTC), No-Feedback group given no gait-related information. Gait stability assessment from plantar pressures. Clinical Tests, Borg Scales, and Falls Calendars: see text. Gait Bio. Baseline gait biomechanics variables for overground and treadmill walking, Fade MTC information reduced progressively for FB group, COPE Centre of Pressure Excursion

To supplement the MTC data, gait kinematics (secondary outcome variables) will be used to assess gait training effects on gait speed, stance/swing times, and joint angles at key events, foot-contact, toe-off, and MTC of the affected and unaffected limb. Baseline and retention clinical data will show how falls risk measures were influenced by feedback training. The association between the clinical tests and the biomechanical variables will be shown by correlating tripping probability and clinical measures. Medio-lateral and anterior-posterior foot pressure (COPE) shifts will reveal biofeedback effects on gait stability. A further secondary outcome is the number of falls following training. These data will be collected via monthly falls calendars for 12 months from enrolment in the study as illustrated in the SPIRIT study timeline (Fig. 2).

### Sample size calculations

In determining sample sizes, for the between-group comparisons, to detect differences in MTC a medium effect size (ES = f = 0.25) between the group effects (No-Feedback versus Feedback) across the two time points (baseline and after training) a total of 126 or 63 per group is required (G*Power [25] calculation, 2 × 2 repeated measures design, α = 0.05, power = 0.85, correlation = 0.7). To allow a 20% drop-out, we shall initially recruit 75 participants per group (*N* = 150). This sample size will also allow detection of an increase of 1 SD in MTC of the biofeedback group, compared to their pre-training. The probability of tripping [7] calculated using the pilot (stroke) participants’ data suggests that a 1 SD (0.77 cm) increase in MTC will result in significant reduction in the risk of tripping on small (1–2 cm) obstacles. A 0.77-cm increase in MTC represents approximately a 50% gain in minimum ground clearance (mean MTC = 1.4 cm) that is shown by our modelling to represent a substantial decrease in tripping risk, i.e. the probability of contacting an obstruction of a given height.

### Data management and statistical analysis

All data will be stored via REDCap, an application specifically designed for the safe storage of clinical research data, for seven years. Only research personnel associated with the study will have access to the data and it will not be available to external agencies. Optotrak, IMU, and Pedar data will be coded so no personal information will be identified in the data. De-identified data will also be stored on a secure drive at the University. The patient’s gait kinematics are monitored as the project progresses to confirm no adverse effects of gait training, such as increased tripping risk. External auditing is not required in this study because all experimenters are trained to identify and record adverse events.

MTC data obtained from the treadmill walking will be analyzed using two intervention group comparisons and repeated measures ANOVA of the intervention groups and change scores, with post-hoc comparisons where relevant. Walking speed will be used as a co-variate due to its influence on MTC data. The between-subject factor will be treatment group (No-feedback and Feedback) with Time (pre-training baseline, post-training, one month, three months, and six months) being the within-subject factor.

1. Between-group comparisons of MTC post-training will reveal effectiveness of the biofeedback gait training method in improving MTC.
2. The within-subject analysis of the Feedback group will reveal the biofeedback effects on MTC by comparing each participant’s after intervention (post-training) data to that obtained in retention conditions, i.e. MTC data at one month (short-term retention) and at three months and six months (long-term retention).
3. MTC data collected during overground walking during retention conditions will determine whether any changes in MTC are translated to overground walking.

## Discussion

This innovative study will evaluate the impact of augmented sensory information for improving gait function, specifically foot-ground clearance, via visually presented biofeedback. The research findings will contribute to the broader falls prevention initiative by demonstrating the effectiveness of toe clearance biofeedback in making walking safer. It will also confirm whether faded feedback enhances learning by demonstrating how performance is affected immediately following training and whether it can be retained in the long term. If shown to be effective, there will be opportunities for biofeedback-training applications to other gait-impaired clinical populations. The project’s findings will contribute to understanding how stroke-related changes to sensory and motor processes influence the tripping risks of everyday walking. The protocol is, however, limited to people with chronic stroke capable of walking on a treadmill due to the continuous real-time feedback used for gait training.

## Trial status

Recruitment began on 27 April 2017 with Protocol Version 6 dated 16 September 2016. Protocol Version 8 dated 20 June 2018 is currently in use following minor amendments not affecting the implementation of the project protocol. Recruitment is expected to be completed by 31 December 2020. Any important protocol modifications will be formally communicated to relevant parties by the chief investigator and the Protocol Version updated.

## Abbreviations

COPE: Centre of pressure excursion
ES: Effect size
FIM: Functional independence measure
FROP-Com: Falls Risk for Older People in the Community
MTC: Minimum toe clearance
RTC: Randomized controlled trial
SD: Standard deviation
